# Supplementary material for: The interactome of intact mitochondria by cross-linking mass spectrometry provides evidence for coexisting respiratory supercomplexes
Source: Mol Cell Proteomics. 2017 Dec 8;17(2):216–32. doi: 10.1074/mcp.RA117.000470 (PMC5795388; doi:10.1074/mcp.RA117.000470)
Supplement: Supplemental Data [file supp_RA117.000470_133922_0_supp_23978_fzffwf.zip › spectra_annotation/mito_spectra_annotation/339-1-32-1-1-1.pdf]

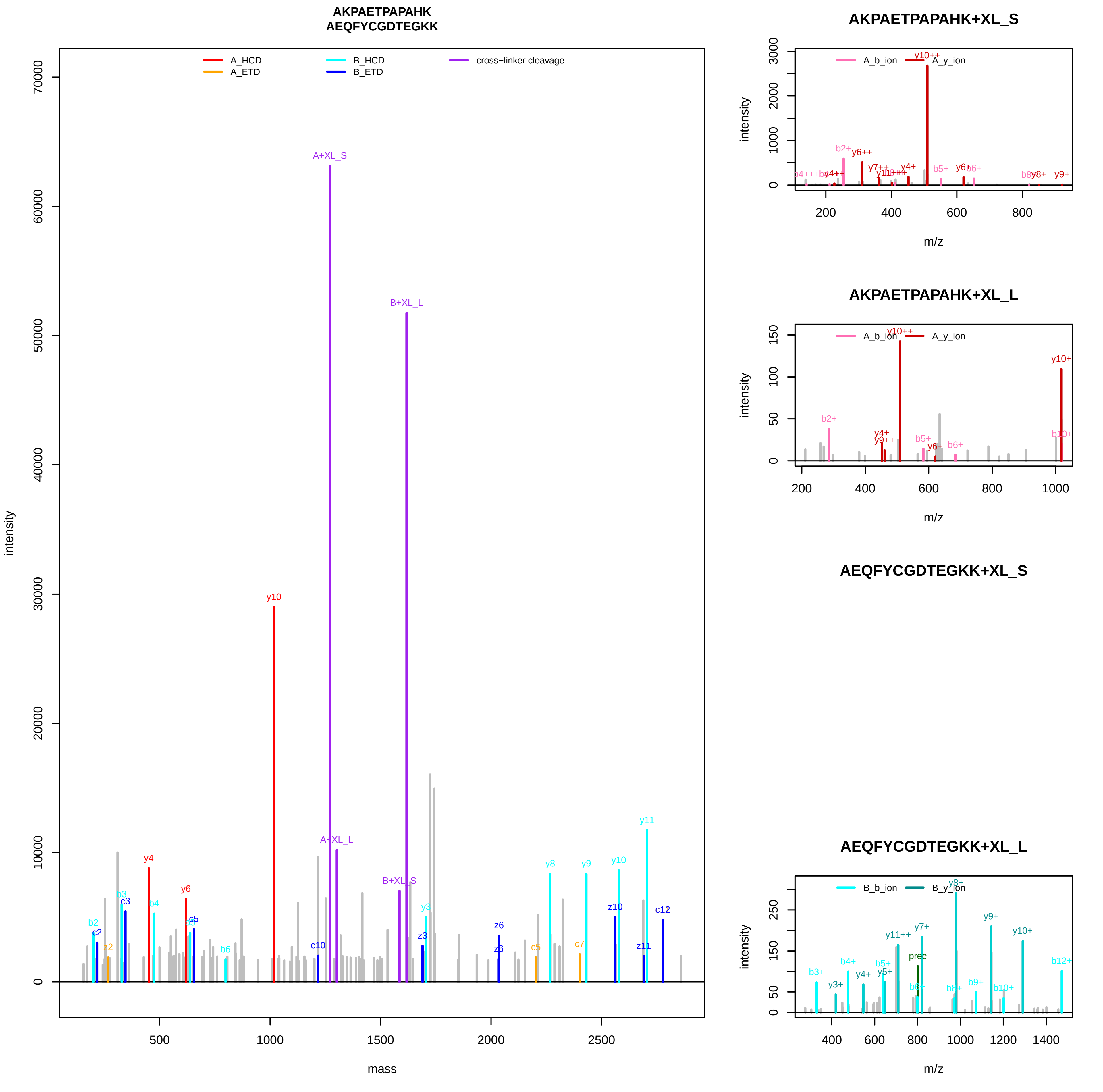
AKPAETPAPAHK
AEQFYCGDTEGKK
A_HCD
A_ETD
B_HCD
B_ETD
cross−linker cleavage
A+XL_S
B+XL_L
A+XL_L
B+XL_S
y10
y4
y6
b2
c2
z2
b3
c3
b4
b5
c5
b6
c10
y3
z3
z6
c5
c7
y8
y9
y10
z10
y11
z11
c12
intensity
mass
0
10000
20000
30000
40000
50000
60000
70000
500
1000
1500
2000
2500
AKPAETPAPAHK+XL_S
A_b_ion
A_y_ion
y10++
b2+
y6++
y7++
y11+++
y9++
y4+
y4++
b5+
y6+
b6+
b8+
y8+
y9+
intensity
m/z
AKPAETPAPAHK+XL_L
A_b_ion
A_y_ion
y10++
y10+
b2+
y4+
y9++
b5+
y6+
b6+
b10+
AEQFYCGDTEGKK+XL_S
AEQFYCGDTEGKK+XL_L
B_b_ion
B_y_ion
y8+
y7+
y9+
y10+
y11++
prec
b3+
y3+
b4+
y4+
b5+
y5+
b6+
b8+
b9+
b10+
b12+
intensity
m/z
